# David Deamer: Five Decades of Research on the Question of How Life Can Begin

**DOI:** 10.3390/life9020036

**Published:** 2019-05-02

**Authors:** Bruce Damer

**Affiliations:** 1 Department of Biomolecular Engineering, University of California, Santa Cruz, CA 95064, USA; bdamer@digitalspace.com; 2 The Biota Institute, Boulder Creek, CA 95006, USA

## Abstract

In commemoration of his 80th birthday, this interview article engages David Deamer in some personal and scientific insights into his fifty year quest surrounding the question of “how can life begin” on the Earth or other worlds.

David Deamer served as editor-in-chief of *Life* from 2014 to 2016. The managing editor of *Life* proposed that the journal should celebrate his 80th birthday this year with a special issue. As a member of the editorial board and his close collaborator over the past nine years, I was more than happy to take on the enjoyable task of telling the story of Dave’s remarkable life in science. The special issue will be introduced with an interview, followed by articles written by his former students and colleagues. I also invited them to contribute vignettes if they cared to, describing what it was like to work with him over the past 50 years.

To put the story in context, I will begin with a brief history. Dave was born on April 21, 1939 in Santa Monica, California. He was named after his father, who was an engineer in the aircraft industry. His mother, Zena, was born in Russia. Her early history has been lost, but a nine-year-old girl named Zenia Meschenk and her seven-year-old sister Weira immigrated to the US on the steamship *Chemnitz* in 1917, accompanied by Helena Mischenk, perhaps to escape the dangers of World War I and the ominous beginning of the Russian revolution. Zena’s parents Stefan and Olga changed their name from Mischenko to Morris in 1906. They were Russian, but may have adopted Zena because there is no record of her birth or citizenship.

WWII stirred up the US like nothing before or since. After Pearl Harbor in December 1941, Dave’s parents felt unsafe in California and moved back to Chicago to be closer to their parents. Dave’s brothers―Richard and John―were born two years apart during this time. When the war ended, the family moved back to Santa Monica and Dave became a student at Beethoven Elementary School. He recalls reading *Rocketship Galileo* by Robert Heinlein when he was 11 years old. His imagination was completely captured by the story of two teenage boys and their uncle, a nuclear engineer, who constructed a thorium-powered space ship that took them to the moon.

In 1952, the family once again returned to the Midwest, finally settling in Westerville, Ohio near Columbus. Dave finished his high school education in Westerville, and demonstrated his early scientific promise in his senior year by being named one of the 40 winners of the Westinghouse Science Talent Search (STS). This was a life changing event because it introduced him as a young scientist just when America was startled by the Russian launch of Sputnik. The US and Russia were already engaged in the Cold War, and were testing hundreds of nuclear bombs for potential use in a possible WW III. Sputnik made the US government wake up to the fact that Americans were in a race with Russians, and that scientists were on the front line. As an STS winner, Dave was featured on the front page of the Columbus Dispatch and was interviewed on a TV program called Imagineering. This was his first taste of fame, but it meant little to him. Instead he just wanted to get on with his college education.

Dave won a scholarship to Duke University, graduating with a Bachelor of Science degree in Chemistry, then completed his PhD in Physiological Chemistry at the Ohio State University School of Medicine in 1965. By this time, he had married, and in 1963 he and his wife Jane had Mark, their first child. Dave knew that he wanted to return to California, so they moved to the Bay Area in 1965 where he accepted a post-doctoral appointment with Lester Packer at UC Berkeley. There he met Dan Branton, a young professor in the Botany department who was pioneering the new technique of freeze-etch electron microscopy. As part of his research, Dave collaborated with Dan to establish how the freeze-etch process worked, and in 1967 they published a cover article in Science. The results confirmed Branton’s proposal that the freeze-etch process actually split membranes down the middle of the lipid bilayer, revealing protein molecules now known to function within the membrane.

In the same year, Dave accepted a faculty position at UC Davis where he soon advanced to associate professor with tenure. Twenty years later, in 1989, he conceived the idea that a single strand of DNA could be drawn through a nanoscopic pore by an applied voltage. If each nucleotide in the strand produced a base-specific effect on the ionic current passing through the pore, the base sequence could be read directly. This concept is now the foundation for a commercial instrument brought to market in 2014 by Oxford Nanopore Technology.

In 1975, Dave spent a sabbatical in England with Alec Bangham at Babraham. His interest in the origin of life was first kindled by conversations with Alec in which they realized that no one had yet thought about the origin of membranes as an essential component of the origin of cellular life. Dave spent the next 40 years working on this question, and in fact the answers he presented are now part of our growing understanding of how life can begin.

Dave’s first marriage ended in 1991, and shortly thereafter he met and fell in love with Ólöf Einarsdóttir who had begun her own career in the Department of Chemistry and Biochemistry at UC Santa Cruz. They traveled to Ólöf’s home in Reykjavik, Iceland for their wedding, after which Dave retired from UC Davis and in 1994 became a research professor at UC Santa Cruz. The couple settled down in the redwood forest of Bonny Doon near the UCSC campus. They have two daughters, Ásta and Stella, now 22 and 18 years old. By a remarkable coincidence, Dave learned that Robert Heinlein had also built his home in Bonny Doon and spent the last ten years of his life there.

Dave continues to be extraordinarily productive during his 25-year tenure at UCSC. He has authored or co-authored more than 200 research articles and reviews that have been cited nearly 30,000 times in the literature. He has also written or edited a dozen books, most recently *Assembling Life* by Oxford University Press [1]. In this scholarly monograph, Dave summarized his research over the past 40 years and invited me to be a co-author of Chapter 10 in which we presented the alternative hypothesis that life did not begin in hydrothermal vents deep in the ocean, but instead arose on volcanic land masses emerging from a global ocean over 4 billion years ago. This hypothesis received a major boost from a field trip Dave and I took in the summer of 2015 when we joined Martin Van Kranendonk, Malcolm Walter, and Tara Djokic to visit the Pilbara region of Western Australia. On the banks of the Shaw River, we gave an impromptu talk to the group in which we sketched out the basic concepts of a fresh water origin set into ancient geological landscapes like the ones we were surrounded by. It turned out that our scenario fit perfectly into Tara’s 2014 Pilbara discovery of geyserite, a mineral laid down by hot water splashing from geysers in hot springs on land. The geyserite contained clear evidence of stromatolites, a fossil imprint of microbial communities. She had discovered the oldest evidence for a hot spring on Earth replete with life 3.5 billion years ago! Our alternative hypothesis of a fresh water origin of life on land which we first presented in [2] was then bolstered by the rock record and presented to the science-interested public for the first time on the August 2017 cover of *Scientific American* [3].

## BD: Was there a single moment in your career when you decided to take up the question of how life could have begun on the early Earth? What was your first experimental work on the topic dating back to your Davis years in the 1970s?

**Figure 1:**
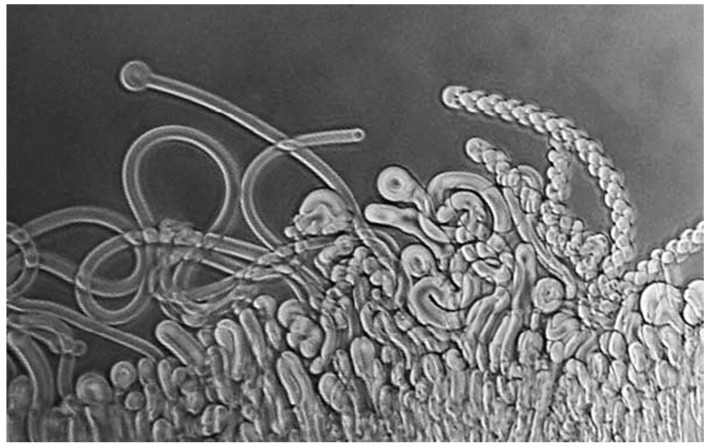
Myelin figures form when a dry phospholipid is exposed to water.

DD: The simplest way to answer your question is to refer to a brief history I wrote a few years back for the journal Astrobiology [4]. During the spring and summer of 1975, I was spending a sabbatical leave with Alec Bangham in his Babraham laboratory, just south of Cambridge, England, when an idea emerged that guided me into research on the origin of life. A few years earlier, Alec had discovered the self-assembly properties of phospholipid and showed that lecithin extracted from egg yolks produced microscopic vesicles when it interacted with water [5]. As the dried lipid absorbs water, it swells and produces wormlike structures called myelin figures (Figure 1). These are unstable, and, given time, ultimately form spherical structures now called liposomes.

Alec also found that the boundary membranes of the vesicles were composed of lipid bilayers that could maintain concentration gradients of potassium or sodium ions. This was the first direct evidence that lipid bilayers were the primary barrier to the free diffusion of ions across cell membranes, a property now known to be essential for all living cells.

**Figure 2:**
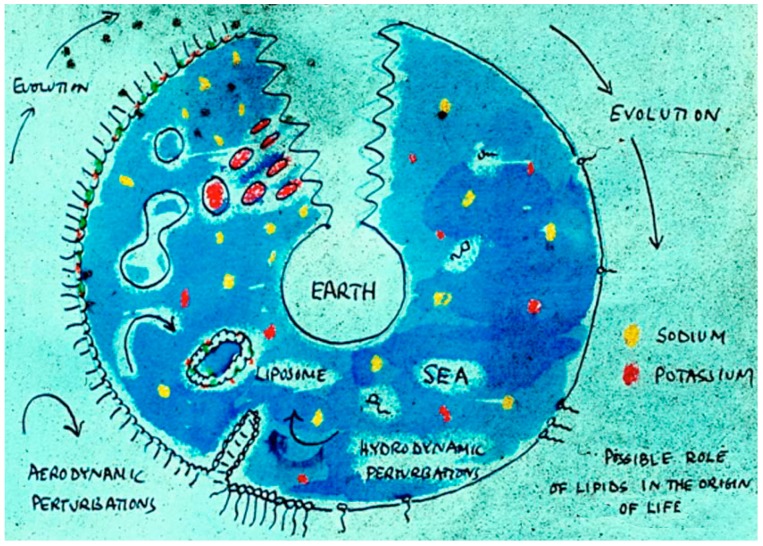
Bangham’s sketch presented in a lecture in Bristol in 1971. It shows the origin of life as self-assembling membranous compartments.

One summer day Alec and I were driving to London in his beloved Morris Mini, and had pulled over to enjoy some cucumber sandwiches prepared by Rosalind Bangham for our journey. The conversation turned to a lecture he had given a few years earlier at Bristol University, with the title “Membranes Came First!” In his talk, Alec argued that the self-assembly properties of lipids were such that membranous vesicles must have been present on the early Earth long before nucleic acids and proteins appeared (Figure 2). But then we were stumped. What was the prebiotic equivalent of lipids, and where did they come from? No one knew.

I took that question home with me when I returned to the University of California, Davis, where I had recently become a tenured associate professor. Will Hargreaves had just joined my lab group, so I posed the question to him as a possible topic for his doctoral research. Will was virtually unique in his ability to be inspired by a challenging question. Over the course of the next few years, Will showed that almost any amphiphilic molecule could form membranes, either by itself or in mixtures with other amphiphiles. Will was able to produce vesicles from molecules as simple as fatty acids, which are little more than hydrocarbon chains with a carboxyl group at one end. Hydrocarbon chains with phosphate or sulfate end groups also worked. Mixtures were often better at self-assembly than pure compounds. For instance, neither dodecanol nor dodecyl sulfate form membranes as pure compounds, but together they produced amazingly stable vesicles. Will was first author on papers in Nature [6] and Biochemistry [7] in which we showed how membranous vesicles could form under simulated prebiotic conditions, fulfilling Alec Bangham’s prophetic claim that membranes came first.

Those papers apparently caught the attention of someone on the panel that reviewed grant proposals for the NASA Exobiology program, and a couple of years later I was invited to become a member of the panel. I have a bit of advice for young investigators. When you are offered membership on a peer review panel, take it! For one thing, you will meet some very smart and talented colleagues. My first panel was chaired by Harold “Chuck” Klein, who was the Biology Team Leader for the Viking Mars landers. A young rambunctious guy named Steve Squyres was also on the panel. Thirty years later, Steve became the principal investigator of the Mars Exploration Rover program, and it was fun to much later see an older rambunctious guy on television, enthusiastically explaining the latest news from Mars.

Another reason to be on a peer review panel is that nothing is better preparation for writing competitive grant proposals than seeing firsthand how proposals are reviewed. It can be disconcerting to discover that a certain amount of luck is involved in getting a proposal funded, but you also learn how to present your ideas clearly, how to argue that they are significant, and how to avoid offending potential reviewers by not citing their research! From my service on the panel, I realized that NASA was a source of funding for just the kind of research I wanted to do, so I wrote my first grant in 1981, it was approved in 1982, and my lab was funded for the next 30 years by the NASA Exobiology and Astrobiology programs.

My involvement with NASA naturally took me to the Ames Research Center in Mountainview, California, which was then and still is a hot bed of science related to what we now call astrobiology. I was invited to give a talk there and met Sherwood Chang, who specialized in meteoritic organics and studied the Murchison meteorite after it fell near Murchison, a tiny village in Australia. The Murchison was composed of a silicate mineral matrix containing over 1 percent by weight of organic compounds. An Ames team led by Keith Kvenvolden had published the first analysis of Murchison organics and convincingly demonstrated the presence of amino acids [8]. I decided to find out if there were other compounds that might assemble into membranes.

**Figure 3:**
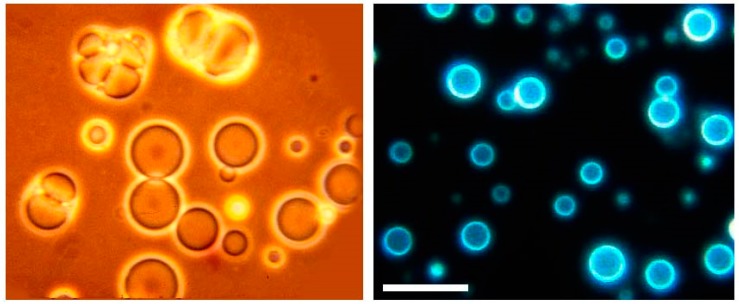
Microscopic membrane vesicles can assemble from amphiphilic compounds isolated from the Murchison carbonaceous meteorite. The compounds are at least 4.5 billion years old, the age of the Earth, and probably older because they were present in the molecular cloud that gave rise to our sun and solar system. The vesicles are fluorescent because they contain polycyclic hydrocarbons like pyrene and fluoranthene. The bar indicates 20 micrometers.

Sherwood gave me a sample to take home to Davis, where I extracted it with a mixture of chloroform and methanol, the solvent we used to extract phospholipids from membranes. I put a drop of the extract on a microscope slide, added water and settled down to watch what happened. It was amazing! I could see the water penetrate into the dried extract, causing round structures to form. A few minutes later I watched thin membranous vesicles begin to ooze away from the surface (Figure 3). I quickly snapped some photographs and made prints. A few of my colleagues in the Zoology department were having lunch down that hall and I barged in to show them my discovery. Alas, they expressed polite interest, then returned to their lunches.

I didn’t have much luck with other scientists when I published the results in Nature [9]. Back then you didn’t count citations to your papers in Google Scholar, but instead counted the number of post cards that requested reprints. I could count these on the fingers of one hand. But the main conclusion slowly sank in, and it seems to be accepted now that membrane-bounded compartments were as essential to the origin of life as amino acids.

## BD: You once described going to visit Peter Mitchell at his unusual home in England. In 1978 Mitchell received the Nobel Prize for the discovery of chemiosmosis. Could you describe meeting him and a bit of his history and the impact his ideas and models have had on the field of origins of life?

**Figure 4:**
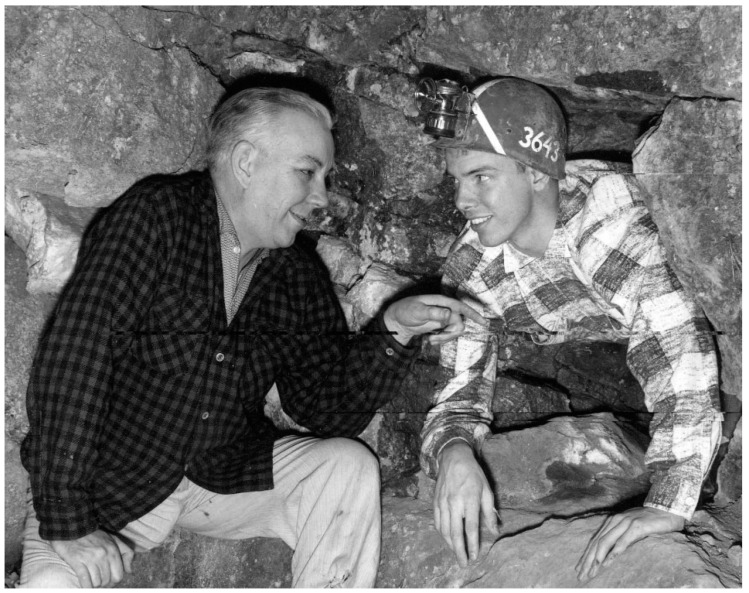
Here is a photo of young Dave (emerging from the rock) and his father. It was taken in 1957 by a photographer from the Columbus Dispatch when Dave was among the 40 winners of the Science Talent Search that year.

DD: In 1971 I was spending my first sabbatical leave at Bristol University in England, working with Tony Crofts. Tony and I had been post-docs in Lester Packer’s lab at UC Berkeley (1965–1967) and became good friends. We went rock climbing in Yosemite, probably one of the most dangerous activities I have ever done, although the cave exploration I did in my late teen years in what is now Mammoth Cave National Park is a close second (Figure 4). Our discoveries of links between three caves were described in a book by Roger Brucker and Red Watson called The Longest Cave.

**Figure 5:**
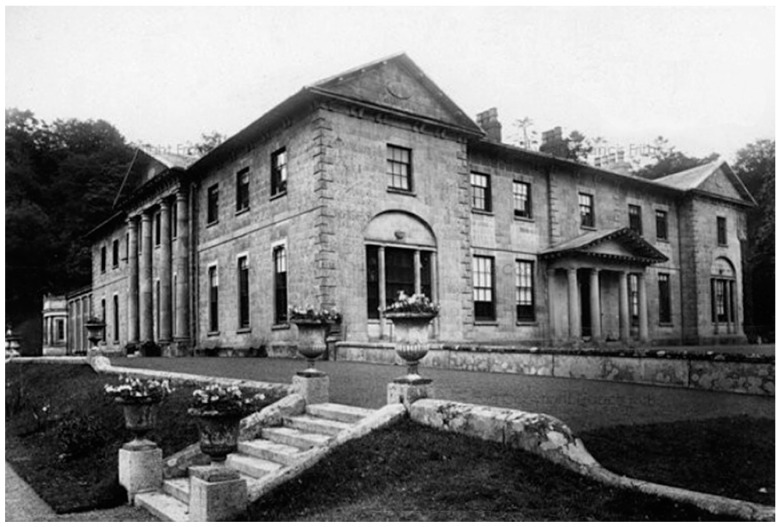
Glynn House, Bodmin in 1897 and Mitchell’s residence from 1964-1992.

**Figure 6:**
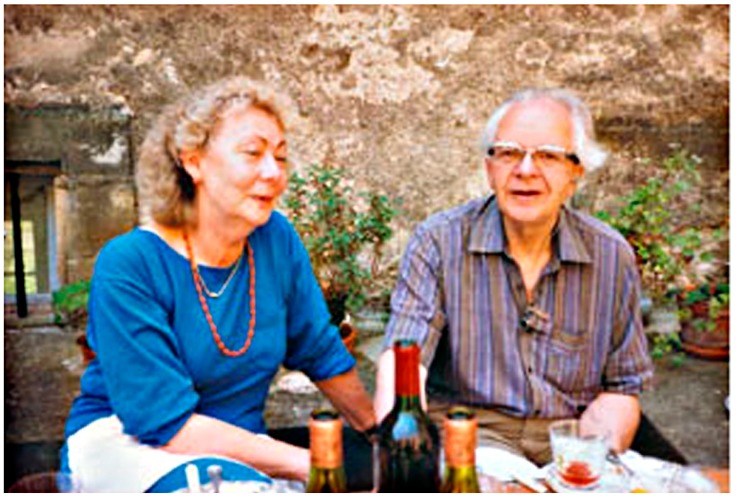
Peter Mitchell and his wife Helen.

Anyway, Tony was an early supporter of Mitchell’s chemiosmosis concept, and our collaboration during my sabbatical was based on how proton gradients across liposome membranes can be produced and then measured with a fluorescent amine called 9-aminoacridine. Halfway through my time in Bristol, Tony suggested that we drive down to visit Mitchell at his private laboratory called Glynn Research. Mitchell and his wife had purchased a Georgian manor house near the tiny village of Bodmin in Cornwall (Figure 5 and Figure 6). Seeing the Glynn House was breathtaking! Imagine having an immense manor house for your very own laboratory. Meeting Mitchell was the first time I had a conversation with someone who would later win a Nobel Prize. It was a revelation to be exposed to a brilliant mind capable of conceiving a unique hypothesis by assembling seemingly unrelated facts into an integrated whole that turned out to be true. If I had to name my role models, it would include Peter, Alec Bangham, Harold Morowitz, and Dan Branton.

## BD: You mentioned that Peter Mitchell was in attendance at the original 1957 meeting in Moscow hosted by early Russian origin of life scientist Alexander Oparin. You showed me the original proceedings of this meeting which included a number of future Nobelists who were there. How did this meeting come about and lead to the formation of ISSOL (the International Society for the Study of the Origin of Life)?

DD: I attended my first ISSOL meeting in 1986 in Berkeley, California, and immediately realized that this group of a few hundred scientists shared my intense interest in the origin of life. I don’t know of any other scientific field that has such a broad scope, ranging from studies of ancient microfossils in the Pilbara region of Western Australia, to simulations of the kind of chemical reactions that could lead to the origin of life on the prebiotic Earth, and to the possibility that life might have begun on Mars. Regarding the history of ISSOL, its origin can be traced back to Alexander Oparin, who published a book in 1928 that was the first serious attempt to understand the origin of life in terms of chemistry and physics and planetary science. Over the next 30 years, he experimented with coacervates, which are microscopic blobs of certain polymers that he thought were models of cellular protoplasm. His reputation as a pioneering researcher grew, and when Miller published his iconic paper on the spark chamber synthesis of amino acids in Science in 1953, Oparin decided that it was time to organize a meeting dedicated to exploring various aspects of the question of how life began. This was the original meeting that led to establishing ISSOL and happened in the same year that I graduated from high school. The following text, slightly edited, is taken directly from the ISSOL website and answers your question about how ISSOL emerged as a scientific society.

“In 1957, the first International Conference of the Origin of Life (ICOL) was held in Moscow, followed by two more meetings, in 1963 in Wakulla Springs and in 1970 in Pont-à-Mousson. During a meeting in 1967 of the Radiation Research Society in Cortina d’Ampezzo, Alexander Oparin, Sidney Fox, Cyril Ponnamperuma, and others discussed the possibility of bringing together those researchers who were studying origin of life initiatives using varied approaches, specifically with the intent of fostering interactions among the international community as an official society. Their idea was to gather the various disciplines under one banner—ISSOL. In 1972, the Society was officially formed and had their first meeting in Barcelona, Spain. The society’s beginning marked a confluence of scientific thought that made the investigation of the origin of life more than just a speculative endeavor: Alexander Oparin’s work on the primordial soup, Sidney Fox’s efforts to understand proteinoids and protocells, Cyril Ponnamperuma’s ideas of chemical evolution, and Stanley Miller’s and Harold Urey’s test of early earth conditions ability to produce organic compounds.

The society grew to sponsor international meetings on a three-year cycle, varying the locales to enable fair access to all of its members. International participation in spaceflight programs in the 1960’s provided an impetus to the growing origin of life community—specifically in the United States—and the society obtained financial support from NASA. Dick Young, the first director of NASA’s Exobiology program, and the program itself, was instrumental in providing direction and funding over the next several decades which supported ISSOL and its members. As manned and unmanned space travel matured, the origin of life research initiatives and interdisciplinary approaches became important to the space community. Real possibilities of detecting life on other bodies in our solar system became an attractive goal. The origin of life field became increasingly interdisciplinary, augmenting its membership with geologists, paleoatmosphere chemists, and astronomers, and the society began reaching out to the wider community, providing a more astrobiological context to its meetings.

The term Astrobiology, though once outside the mainstream of scientific inquiry, was a formalized field of study as early as 1960. In 1998 NASA established the Astrobiology Institute to perform research in astrobiology which increasingly became a global endeavor with partners in Europe, Australia, Spain and England. In 2005 at the ISSOL meeting in Beijing, ISSOL determined that developments in origin of life research and the maturing Astrobiology discipline, provided an overlap in interest that needed to be reflected by ISSOL. The society voted and adopted the new name ISSOL—The International Astrobiology Society.”

## BD: Your involvement with ISSOL has been long, and you served as its president in the years 2011–2014. ISSOL has had some legendary meetings in which curious characters engage in quite heated debates. I recall reading about one such meetings where they almost came to fisticuffs. Can you recall some key moments of interest at these meetings?

DD: I don’t recall anything like that at ISSOL meetings, but two other conferences are also forums for origins of life research. There is a Gordon Conference dedicated to origins of life, and AbSciCon (Astrobiology Science Conference) is a conference sponsored by the NASA Astrobiology Institute. You might be thinking of the 2002 AbSciCon that was organized by Lynn Rothschild at the NASA’s Ames Research Center. In his book *Gen.e.sis*, Bob Hazen described a confrontation between Bill Schopf (UCLA) and Martin Brasier (Oxford University). Bill had been among the first scientists to obtain samples from the 3.4-billion-year-old Apex Chert formation in the Pilbara region of Western Australia, the same site you and I visited in 2015. Bill made thin sections of the chert minerals and observed structures resembling fossilized microbial life. He published the images in Science, and for many years these were accepted as evidence for the earliest known life. Brasier became interested and asked to see the thin sections. However, his conclusion was that these were not bacteria, but simply artifacts produced when non-biological organic material was squeezed into cracks in the mineral matrix. The AbSciCon meeting was the first time the two met in public, and in his book, Hazen described it this way: “*As Brasier calmly outlined his arguments, the scene on stage shifted from awkwardly tense to utterly bizarre. We watched amazed as Schopf paced forward to a position just a few feet to the right of the speaker’s podium. He leaned sharply toward Brasier and seemed to glare, his eyes poring holes in the unperturbed speaker… Perhaps Schopf was just trying to hear the soft-spoken Brasier in the echoing hall, but the audience was transfixed by the scene… Afterwards many of us breathed a sigh of relief that no blows had been exchanged, and then we tried to figure out who won.*”

## BD: What led you forty years ago to take the brave step of leaving the clean controls of the lab and explore prebiotic chemistry in messy and sometimes dangerous locales such as in the hydrothermal fields of Bumpass Hell here in Northern California, and those in distant Kamchatka, Russia? What do you think these excursions have done to open your understanding to conditions on the early Earth and how to bring those conditions back to your laboratory simulations?

DD: Our family moved to Davis, California in 1967 when I accepted a faculty position in the Zoology department at the university. In June 1970 my wife and our son were visiting relatives back in Ohio and I decided to explore Mt. Lassen National Park while they were away. There had been a lot of snow that year, but the road into the park had been recently plowed and I remember driving uphill between snow banks over 6 feet high. The cleared portion of the road ended at a parking lot with the curious label of Bumpass Hell. What could that be? No one else was there, so I took my first hike on a snow-covered trail that is now very familiar. After half an hour I began to smell the sulfur aroma characteristic of volcanic hot springs and could see clouds of steam billowing into the cold air. I finally hiked down the last steep portion of the trail and there it was, Bumpass Hell… impressive! Hot springs and a boiling lake had melted several acres of the snow that covered the surrounding area. I walked closer to see one of the boiling pools up close, and just like the 19th Century guide who lost a leg there and gave his name to the site, my foot broke through the snow. Mr. Bumpass fell into boiling mud three feet deep and lost his leg, but fortunately I only broke through three inches, still enough to have boiling water get over the top of my boot and give me a painful burn. I limped back to the car and drove home, my curiosity satisfied.

For the next thirty years I gave little thought to volcanoes because I was busy in the laboratory working on liposomes and proton transport. But then I met Vladimir Kompanichenko, a very friendly volcanologist from Khabarovsk in far eastern Russia who visited Santa Cruz in 1999. Vladimir told me about some amazing volcanic regions in Kamchatka and mentioned that organic compounds had been detected in some of the hot springs and pools, particularly the Uzon crater which had a film of oil floating on the surface of a lake there. Well, THAT was interesting! What if the organics were actually being synthesized by abiotic chemistry? This would be a major discovery vindicating that biologically relevant organic compounds could be synthesized geochemically in volcanic conditions. I decided to take Vladimir up on his offer and became the U.S. organizer for field work there.

Barry Blumberg was the new director of the Astrobiology Institute at NASA Ames, so I wrote to him directly, telling him about Kamchatka and requesting funds for a field trip. Barry liked the idea, came up with travel funds for us, and in 2001 our little party flew up to Anchorage Alaska, then next morning clambered into an ancient Magaden Airlines jet that flew once a week to Petropavlovsk. Our group included Sherry Cady, Chris McKay, John Spears, and Jonathan and Suzanne Trent. During the trip we flew by helicopter to volcanic peaks and drove to Mount Mutnovski, where we hiked down into the crater of an active volcano. This was my first experience with what we now refer to as a prebiotic analogue site. In other words, it was a world dominated by lava, boiling water, and billowing steam, with no visible life except our little group of scientists. This visit had been so compelling that I decided that we had to return, and in 2004, we were funded by the NASA Astrobiology Institute for a second and more ambitious trip.

Because we had detected no organic compounds in the water samples brought back from the first field work on Mutnovsky, this time I took along a mixture of amino acids, nucleobases, phosphate, glycerol, and myristic acid that we knew could assemble into membranous vesicles. On the fourth day of the trip, we visited a hydrothermal outbreak on the side of Mutnovsky that resembled Bumpass Hell. An isolated pool of boiling water there, probably not the “warm little pond” that Darwin envisaged for life’s beginnings, but instead a “hot little puddle”. It was ideal for the experiment I had in mind, so I dumped in the mixture and sat down to wait and see what happened over the next few hours.

**Figure 7:**
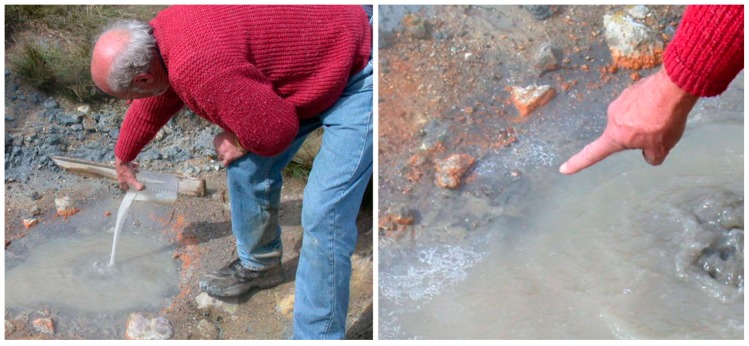
Adding a “prebiotic soup” to Darwin’s hot little puddle at Mt. Mutnovsky, Kamchatka, Russia. A white, membranous froth immediately appeared around the edges.

I didn’t have to wait long. Within a few minutes, a white froth appeared around the edge of the puddle (Figure 7 below). That simple observation inspired all the research that followed, because I knew that the froth was composed of membranous vesicles of myristic acid, and that the vesicles had captured samples of the other compounds in the mixture. As the pool evaporated over time, the vesicles and their contents would fuse into a thin film of organics on the mineral surfaces. Most important of all, if the organics were monomers such as amino acids or nucleotides, it was possible that they would condense into polymers during the drying process. Then when it rained or the pool refilled from the hot spring and the film was rehydrated, I knew from my previous work that the polymers would be encapsulated within the vesicles, the first step toward cellular life.

## BD: As was mentioned earlier, we had the opportunity to journey to Western Australia to join a group led by University of New South Wales geologists Malcolm Walker and Martin Van Kranendonk on an Astrobiology field trip. I recall our sense of wonder as we touched the tops of living stromatolites in Shark Bay and only days later traced our fingers along the layered fossil ridges of their ancient forebears over three billion years earlier. What insight and impact did this trip have for your lifetime of work in the origin of life?

DD: I had been hearing about Malcolm Walter for years and the two of us had recently been in email communication about a possible ISSOL meeting in Cairns, so it was a pleasure to meet him in person. Martin Van Kranendonk was new for me, as was his PhD student Tara Djokic, so it was one of those wonderful serendipities that you and I happened to meet all three of our future colleagues on that trip.

**Figure 8:**
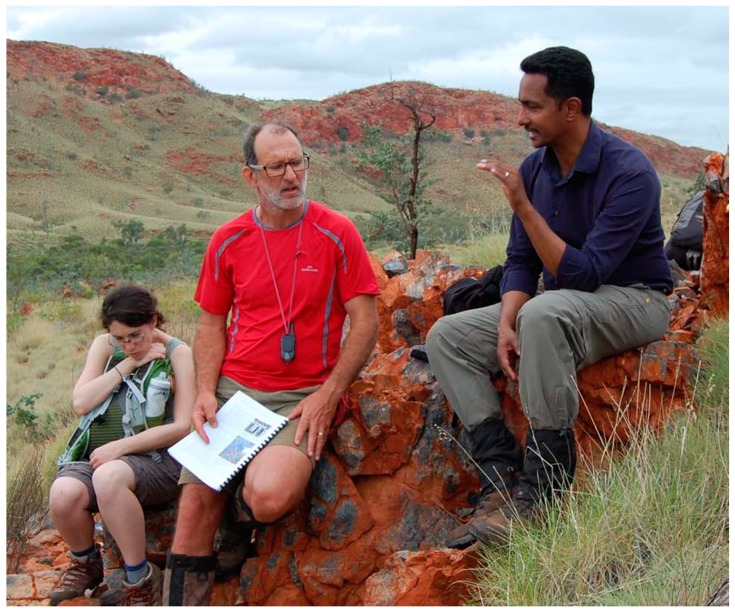
Martin Van Kranendonk and Ray Jayawardhana have a conversation, presumably inspired by sitting on a rock formation over 3 billion years old.

The first portion of our trip was a plane flight from Perth to Carnarvon, and from the airplane we could look down on Shark Bay, where we would later see and touch living stromatolites. At the airport, our party of 24 clambered into the vehicle that would be our home for the next ten days. On the two-day drive north we made stops at the Tom Price iron mine and Karajini National Park, then finally arrived at the amazing Pilbara craton, where we hiked in the Tumbiana region with its “young” stromatolites (only 2.7 billion years old), clambered up to the Dresser formation, and journeyed onwards to the Apex Chert where 3.4 billion year old microfossils were discovered by Stanley Awramik and Bill Schopf, and more recently confirmed by Martin Brasier. Here is a photo of Martin (red shirt) exchanging details of the rock record with Ray Jayawardhana of York University on a cliff called Knossus in the Tumbiana (Figure 8).

**Figure 9:**
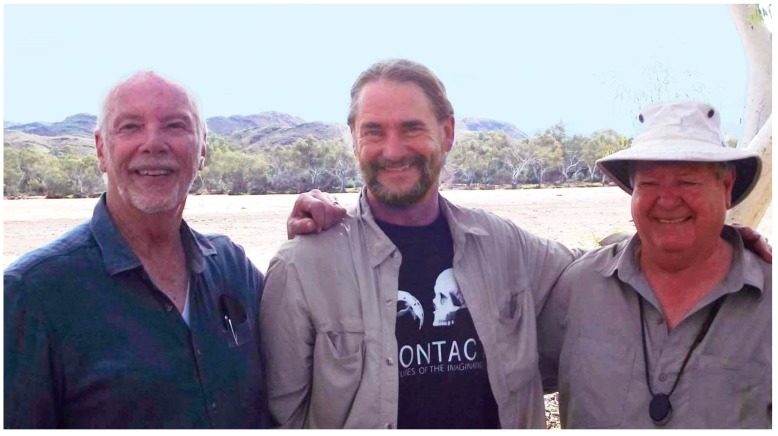
Dave, Bruce and Malcolm on the banks of the Shaw River.

At the end of our trip we camped on the Shaw River next to the Strelley Pool Formation, the original stromatolite discovery outcrop. Malcolm invited us to give an informal talk to the captive audience, and we were happy to oblige. During the talk we explained the hot spring hypothesis that you and I had been developing and how it fit into what we had seen in Pilbara. That talk rang a bell with Martin, because his student Tara had recently discovered evidence of the mineral geyserite in the Dresser formation, which can only form in conditions associated with fresh water hot springs like those in Yellowstone. In 2017, Tara was first author on a paper in Nature Communications [10], and Martin was first author of an article in Scientific American in which we had a chance to explain the hot spring hypothesis for public consumption [3]. Here is a photo of me (left), you (center), and Malcolm (right), standing on the banks of the Shaw River that day (Figure 9).

## BD: Schopenhauer said that “All truth passes through three stages. First, it is ridiculed. Second, it is violently opposed. Third, it is accepted as being self-evident.” Over the years you have seen your ideas and experimental results demonstrating nonenzymatic polymerization through wet–dry cycling in the presence of lipid go through some of these stages. Yet, many of our colleagues do not yet take it as self-evident that dehydration is the sole available and necessary path to the generation of lengthy polymers needed for life to start. How have you managed to stay optimistic through this long process, and what is your feeling about what it will take to convince more of our colleagues of the viability of this approach?

DD: The answer is evidence, evidence, and more evidence until our colleagues decide to try it themselves, realize that it actually works, and furthermore that they do not have a feasible alternative energy source providing a pathway to polymerization [11]. I like to think that life either began in salty seawater, as most people believe, or in fresh water hot springs, as we have argued. That gives us a 50/50 chance of knowing how life can begin, and those are pretty good odds for research on such an important question! Little by little, it seems to be sinking in. For instance, Nick Hud incorporated wet–dry cycles in a few of his experiments [12], and Maikel Rheinstadter at McMaster University constructed the world’s most sophisticated simulation chamber that can test some of the predictions of the hypothesis [13].

## BD: You invented the technique that led to today’s nanopore sequencing revolution and products like the MinION and PromethION from Oxford Nanopore Technologies. This history has been documented in a number of recent articles [14]. What is your plan and hopes for your current venture, UpRNA?

**Figure 10:**
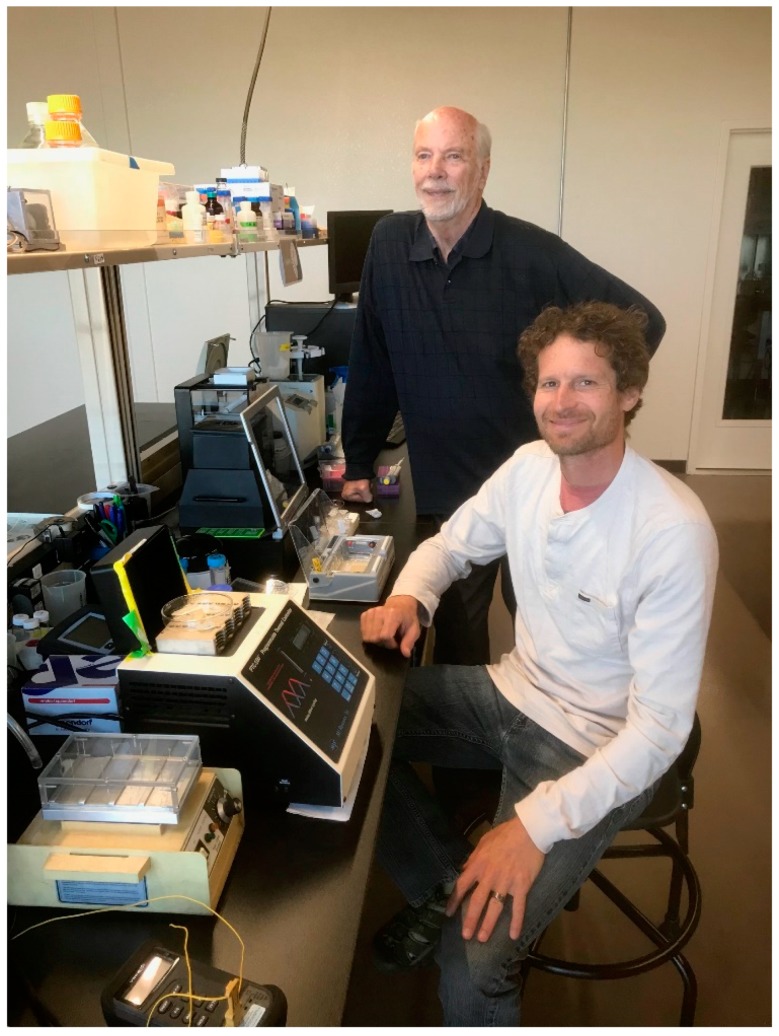
Dave and Gabe Mednick at UpRNA in Santa Cruz.

DD: It is very satisfying to me that we have found a way to bring together two major lines of my research: the origin of life and nanopore sequencing. I am a founding partner for UpRNA, a startup company here in Santa Cruz which has the goal of transcribing base sequences from short DNA templates to RNA oligomers called siRNA (silencing RNA) which could be instrumental in fighting viral infections (Figure 10). In other words, we are taking what I have learned about the origin of life and testing it as a spinoff for making a potentially valuable pharmaceutical agent. We will use the MinION nanopore sequencing device for quality control.

## BD: Considering all of your life’s work on where life can begin, what is your best guess of how replete the cosmos is with simpler, microbial life, and how common might more complex forms be which became advanced enough to look back and consider the question of their own origins?

DD: I just got back from UC Berkeley, where I attended a conference hosted by the Breakthrough Initiative. The theme of the conference was Migration of Life in the Universe, which is exactly your first question. Of course, the century-old concept of panspermia was brought up, the idea that there is life throughout the universe which was delivered to the early Earth at the end of accretion after the ocean had formed. In these enlightened times, the audience decided that panspermia was no longer appropriate and is attempting to come up with a better term. Quite a few were proposed. I also thought about the question and realized that in the absence of evidence, we should also have some terms that describe what we do know for sure. The ones I came up with include *panhydria* (water is everywhere) *pancarbonia* (carbon is everywhere), and *panorganica* (organic compounds are everywhere.) So, given those facts and what we have discovered, a reasonable assumption is that microbial life will quickly emerge on habitable planets like the early Earth and Mars. Of course, on a geological time scale “quickly” could mean 100 million years!

Advanced life is another question entirely. Just consider all the hurdles that must be overcome: photosynthesis must begin so that atmospheric oxygen becomes available. Given oxygen, eukaryotic cells can emerge. At some point eukaryotes become multicellular and divide into autotrophic life (plants) and heterotrophic life (animals that eat plants). Certain animal cells form a sensory nervous system, and others become a motor system that controls movement. As predator–prey relationships begin and the environment becomes ever more complex, there is selective pressure toward ever increasing size of the central nervous system. The result is that brains become anatomical features. Finally, when the cerebral cortex of primates reaches a certain size, say ten billion nerve cells, with synaptic connections per cell numbered in the tens of thousands, self-awareness and consciousness emerge, and that is where evolution has taken us so far. My point is that every one of those hurdles must have been overcome for human consciousness to be possible! This seems very iffy to me, so there may be many planets in the galaxy stuck with just microbial life, as life on Earth was for over two billion years, but only a few with intelligent life. 

## BD: As you guide the next generation of young investigators and assist new laboratories to set up their experimental simulations and field work, what is your best-case scenario for the state of the inquiry into the origin of life a decade or two from today?

DD: I would guess we know maybe 1% of what is necessary to understand how life can begin. The other 99%… well, wherever you look in origins of life research, there are vast gaps of ignorance that are within the reach of anyone who wants to try their hand. I identified some of these gaps in Chapter 11 of my book, *Assembling Life* [1]. For example, how did life become homochiral? How were polymers synthesized non-enzymatically for life to begin? How did metabolism begin? How was light captured in primitive versions of photosynthesis? Where did ribosomes come from and how did the genetic code emerge? As David Cornwell, my PhD advisor used to tell me, quoting from the Book of Matthew, “The harvest is plentiful, the workers are few.”
